# Impact of physical exercise in advanced‐stage cancer patients: Systematic review and meta‐analysis

**DOI:** 10.1002/cam4.4746

**Published:** 2022-04-11

**Authors:** Sergio Rodríguez‐Cañamero, Ana Isabel Cobo‐Cuenca, Juan Manuel Carmona‐Torres, Diana Patricia Pozuelo‐Carrascosa, Esmeralda Santacruz‐Salas, Joseba Aingerun Rabanales‐Sotos, Tatiana Cuesta‐Mateos, José Alberto Laredo‐Aguilera

**Affiliations:** ^1^ Grupo de Investigación Multidisciplinar en Cuidados (IMCU), Universidad de Castilla‐La Mancha Toledo Spain; ^2^ Clínica Hemodiálisis Avericum Toledo Toledo Spain; ^3^ Facultad de Fisioterapia y Enfermería de Toledo Universidad de Castilla‐La Mancha (UCLM) Toledo Spain; ^4^ Department of Nursing, Physiotherapy and Occupational Therapy, Facultad de Enfermería Universidad de Castilla‐La Mancha Albacete Spain; ^5^ Grupo de Actividades Preventivas en el ámbito Universitario de Ciencias de la Salud (GAP‐CS) Universidad de Castilla‐La Mancha Albacete Spain; ^6^ Servicio de Urgencias del Hospital Universitario de Toledo (HUT) Toledo Spain

**Keywords:** cancer, cancer patients, metastasis, physical exercise, quality of life

## Abstract

Health professionals predict that the number of people who will suffer and die from oncological diseases will continue to increase. It is vitally important to provide comprehensive care to these patients and prescribe physical exercise programs as adjuvant therapy. The objective of this systematic review was to determine the impact of physical exercise on advanced‐stage cancer patients. A literature search was performed in eight different databases. This search focused on randomized controlled trials (RCTs) published during the last 10 years. To assess the methodological quality of the sample of 15 RCTs finally obtained, the PEDro scale was used. Aerobic and strength training methods were used. The combination of both aerobic and strength training methods was the most frequently reported. Likewise, different physical and psychological variables were recorded, from which improvements were seen in fatigue, independence, quality of life and sleep, among others. The participation in physical exercise programs by advanced‐stage cancer patients has a positive impact on health. Providing these programs serves as adjuvant therapy, facilitating the comprehensive care of patients. Similarly, aerobic, strength or mixed training programs increase the muscle mass of patients and therefore reduce hypotonia, the main side effect during the advanced‐stages of cancer.

## Graphical abstract

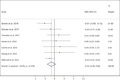

## 1 INTRODUCTION

Cancer is the main cause of noncommunicable disease mortality.^1^, ^2^ Regarding etiology, the factors that trigger it can be social determinants, genetic factors, environmental exposures, chronic infections, and lifestyle.^3^, ^4^, ^5^, ^6^, ^7^

Because life expectancy has increased^8^ and cancer may be derived from the failure or deterioration of a person's body, it would be reasonable to expect that the number of people suffering from this disease is increasing. Therefore, risk factors, prevention and early diagnosis of diseases that cause cancer have become objectives of the scientific community.^9^ Studies have identified factors that can reduce the risk of developing cancer and provided guidance regarding improving modifiable risk factors, including sedentary lifestyle, smoking, alcoholism or an inadequate diet, among others. For these modifiable risk factors, it has been proven that physical exercise acts as a protective factor and reduces the chances of developing cancer.^9^

According to the Spanish Society of Medical Oncology (Sociedad Española de Oncología Médica, SEOM), worldwide, in 2018, more than 18 million people suffered from cancer, with an estimated 30 million people projected by 2040.^10^ Regarding mortality in Spain, cancer currently causes more than 27% of total deaths, being the leading cause of death in men and the second in women.^11^ Similarly, according to organizations and professionals in the sector, that percentage is estimated to increase to 71% or higher by 2040.^10^ As a result, this increase would mean an increase from approximately 9 million deaths worldwide in 2018 to more than 16 million in 2040.^10^, ^11^

When estimating the economic impact that cancer has on the Spanish National Health System (Sistema Nacional de Salud Español, SNSE), it currently represents more than 0.66% of the gross domestic product (GDP), that is, more than 7000 million euros per year.^11^ Therefore, one of the most efficient ways to reduce health spending, following the recommendations of the literature, would be to include physical exercise programs within health policies.^12^, ^13^ Thus, efforts should be directed toward prevention to delay the age of onset of cancer as well as to reduce modifiable risk factors, guaranteeing a higher quality of life for the population.^12^, ^14^ In fact, in addition to directly affecting the health of the population, it has been shown that each euro invested in promoting and participating in physical exercise represents a savings of fifty euros in health; such an investment would achieve economic savings goals as well as unburden the SNSE.^12^

The classification of cancer diagnoses ranges from stage 0 (asymptomatic) to stage IV (terminal, with continuous progressive deterioration).^14^, ^15^, ^16^ The American Cancer Society classifies patients who have stage III and IV in an advanced‐stage, understanding them as terminal/metastatic and with a worse prognosis.^17^ In the case of stage III, the tumor has already spread to other lymph nodes far from the original location of the tumor and has even invaded other tissues near the tumor.^17^ On the other hand, in stage IV, there has already been a metastasis of the tumor to other organs of the body.^17^This detriment and wear on the health and physical qualities of cancer patients manifest as increased fatigue, muscle atrophy, loss of physical condition, paresthesias, weakness, and deterioration of quality of life, among others.^14^ All these negative effects deteriorate the quality of life of patients, even more so in those who are in an advanced‐stage; however, the deterioration can be avoided with physical exercise.^14^

The main therapeutic approaches for treating patients in stage III or IV cancer involve, to a large extent, pharmacology, surgery, chemotherapy, radiotherapy or radioiodine therapy, with no other auxiliary therapy.^18^, ^19^, ^20^ These conventional therapies, in turn, have been shown to significantly decrease quality of life, in addition to causing other side effects, such as fatigue, weight loss, diarrhea, hypertension (HT), and alopecia.^18^, ^21^ Of these secondary factors, weight loss is one of the most widespread.^22^, ^23^ This side effect leads to a decrease in patient muscle mass, which is associated with a loss in independence and autonomy, thus affecting quality of life.^22^, ^23^ Similarly, it is important to emphasize that physical exercise, in a healthy population, increases muscle mass, decreases fat, improves physical appearance, increases vitality, has relaxing effects and facilitates socialization, factors that reaffirm the need for physical exercise as adjuvant therapy.^24^

It should be noted that physical exercise is defined as the regular, planned, structured, and repeated practice of physical activity, with the aim of improving the physical condition of the subject, in which parameters of intensity, volume, frequency, and types of sports disciplines are taken into account.^25^, ^26^ Nowadays in patients who have cancer, physical exercise has been implemented as an auxiliary therapy in early or asymptomatic stages.^27^ In contrast, there is no such demand or use of physical exercise as adjuvant therapy for people with advanced‐stage cancer. However, recent studies have shown how the use of physical exercise, as an adjuvant therapy, has benefits for people with advanced‐stage cancer or metastasis.^27^ These benefits are evidenced as lower stress, anxiety, and depression as well as improvements in pain, fatigue, respiratory distress, constipation, and insomnia in addition to ensuring patient safety during interventions, but still no physical exercise guidelines have been indicated to obtain benefits such as type of training, volume, frequency, and intensity..^13^, ^28^, ^29^, ^30^, ^31^, ^32^

Considering the deterioration produced by cancer together with the side effects caused by pharmacological treatments, the main objectives of this review are to determine the impact that physical exercise has on advanced‐stage cancer patients and to identify the physiological benefits of physical exercise in advanced‐stage cancer patients and quantify the level of physical exercise suitable for patients in advanced‐stages of disease.

## 2 METHODOLOGY

This is a systematic review that was conducted based on Preferred Reporting Items for Systematic Reviews and Meta‐Analyses (PRISMA).^33^ In addition, this systematic review was registered and published in Prospero with the ID: CRD42021268636.

### 2.1 Search strategy

A comprehensive electronic search was conducted from November 30th, 2020 to January 31th, 2021 in the following databases: Cochrane; CSIC; EBSCOhost; ProQuest; PubMed; SciELO; Scopus and Web of Science.

The search strings used different health sciences descriptors (DeCS) and medical subject headings (MeSH), together with the following Boolean operators: “AND” and “OR”.

Table 1 shows the PICO criterion. Similarly, each PICO criterion is related to the search string, following the reference words used for each of them.

**TABLE 1:** PICO criteria

Criteria (PICO)	Keywords
Patient (P)	(Advanced cancer OR Metastasis OR Terminal illness) AND (End of life OR Palliative care)
Intervention (I)	Physical Activity OR Exercise OR Physical exercise
Comparison (C)	Control Group OR Intervention Group (Physical exercise) Aerobic Group OR Strength Group
Outcome (O)	Improvement in physical condition

### 2.2 Study selection

After eliminating duplicate articles, two reviewers (SRC and JALA) independently and blinded to one another assessed the titles and abstracts. Afterward, the reviewers evaluated the full text of selected articles. If any discrepancy arose between the 2 independent reviewers, a third reviewer (JMCT) was consulted.

The inclusion criteria were as follows: (I) articles and/or studies published in the last 10 years, from 2011 to 2021; (II) patients who had advanced cancer (stages IIIA or B and IV) at the time of the intervention, without distinction of gender, sex or age; (III) studies that specified the intervention and/or physical exercise program as an adjuvant therapy to the main therapy; (IV) randomized controlled trials (RCTs) and nonrandomized clinical trials; and (V) articles written in English or Spanish.

The exclusion criteria were the following: (I) studies with samples composed of people with cancer in a non‐advanced‐stage or who have overcome the disease; (II) articles outside the established publication period; (III) studies that did not specify the type of intervention or did not quantify the physical exercise performed; (IV) studies that used programs that were not prescribed and controlled by a professional; (V) studies that were not performed in humans; and (VI) studies with a score lower than 8/10 on the PEDro scale, because with this score is when studies are considered, methodologically, as a very good or excellent quality.^34^

### 2.3 Evaluation of the quality and evidence of the studies

To maximize the quality of this study and following the standards set for systematic reviews, we evaluated the quality and internal validity of the different studies chosen. To this end, a critical analysis of the studies (all were RCTs and nonrandomized clinical trials) was performed using the PEDro scale. This scale comprises 11 items, among which the mode of determining sample eligibility, the blinding of the sample or researchers, the random distribution of the sample, and the dropout rate are evaluated.^34^ For the evaluation of the quality of the RCTs, the literature establishes a grade below 4 as poor, between 4 and 5 as fair, between 6 and 8 as good and above 9 as excellent.^34^

To ensure the highest quality for this review, the GRADE (Grading of Recommendations, Assessment, Development, and Evaluation) and SIGN (Scottish Intercollegiate Guidelines Network) systems were applied to analyze the methodological quality and design of each study. Based on the GRADE system, all the RCTs were considered of the highest quality because they had a high level of evidence. Based on the SIGN system, the studies used in this review were RCTs or nonrandomized clinical trials with a high level of evidence (+); studies with strong evidence present a low risk of bias.

### 2.4 Data synthesis and analysis

**FIGURE 1:**
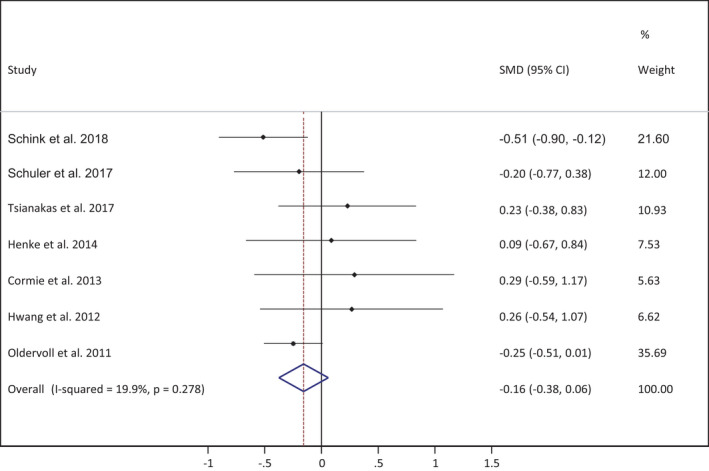
Meta‐analysis for the fatigue variable

The effect size of each study was calculated as the standardized mean difference (SMD) in fatigue (Figure 1). The effect size of the parameters from pre‐ to post‐intervention between groups (exercise intervention vs. control)^35^ in each study were calculated and pooled using the random‐effects model (DerSimonian–Laird approach), assuming a correlation coefficient of 0.5.

Finally, the effect sizes of all studies included were combined to estimate an overall summary effect size, with a 95% confidence interval (CI) and a random‐effects model.

Study heterogeneity was assessed using the I2 statistic, and the following values were used for interpretation: 0% to 40% might not be important, 30% to 60% moderate heterogeneity, 50% to 90% substantial heterogeneity and 75% to 100% considerable heterogeneity; the corresponding p‐values were also taken into account.^36^

To analyze the influence of each study on the overall ES, a sensitivity analysis was conducted. For this, each study was deleted from the model, and the pooled analysis recalculated.

To test publication bias the visual inspection of funnel plot and the Egger test were used.^37^ Significance was set at 0.05.

Statistical analyses were performed using STATA® SE software, version 16 (StataCorp, College Station, TX, USA).

## 3 RESULTS

From the 8 databases used for this systematic review, a total of 571 results were obtained. Following the criteria established for the search, a total of 556 studies were eliminated from the sample.

**FIGURE 2:**
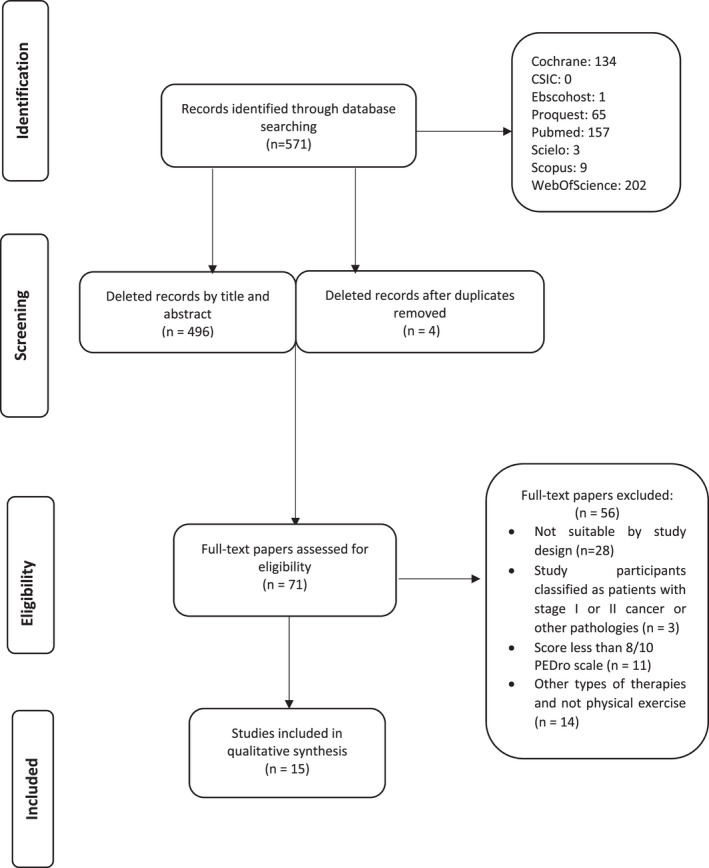
PRISMA flow diagram

Following the PRISMA selection criteria (Figure 2), the final sample for the development of this systematic review consisted of 15 randomized and nonrandomized clinical trials.

The 15 studies included in the final sample met the proposed inclusion criteria. All the included studies were written in English or Spanish in the last 10 years, presenting a randomized or nonrandomized clinical trial design in which all physical exercise interventions were specified and all included patients had advanced‐stage of cancer at the time of the intervention.

The descriptive characteristics of the sample reflect a total of 1072 patients, of whom 60% were women and 40% were men. The cancers most commonly addressed in the different interventions were heterogeneous tumors (50%), breast cancer (22%), lung cancer (18%), colorectal cancer (6%) and prostate or gastrointestinal cancer (2%). Notably, this sample was distributed across different countries, such as Germany, the United States, Australia, Taiwan, Great Britain, Norway, and Poland.

The final sample was obtained following the criteria of physical exercise as an adjuvant therapy to the main therapy in the different interventions. This therapy of prescribed and scheduled physical exercise, such as aerobic and strength training, stands out for its requirements for proper supervision by physical exercise/rehabilitation specialists as well as by a multidisciplinary team.

Table 2 provides the most important data of the selected studies.

**TABLE 2:** Results table

Authors (year)/Country	Design (Cancer)/Participants	Intervention	Primary Outcome	Conclusions	Quality
Rutkowska et al.^38^ (2019) Poland	RCT *n* = 30; Lung cancer. Intervention Group (IG) n = 20; 59.1 ± 6.8 years. Control Group (CG) *n* = 10; 61.3 ± 8.8 years.	Duration: 4 weeks. Description: physical exercise programme while the sample received cycles of chemotherapy and cytostatic drugs. It focused on aerobics, strength, and Nordic walking. 5 supervised training sessions were carried out per week, each lasting 60 min.	Significant increase for IG in the 6‐min walking test (6MWT), standing up and walking test (*p* = 0.01). Likewise, the IG registered a significant increase in the getting up from a chair and flexing arms tests (*p* = 0.001). Spirometry values significantly improved for the group that participated in the intervention: Fev1 (*p* = 0.01) and Fev1 / Fvc (*p* = 0.04)	Regulated, supervised and planned physical exercise in patients with advanced lung cancer during chemotherapy treatment has a beneficial effect on mobility and physical fitness. Regarding spirometry values, physical training proves to be beneficial for FEV1, FVC and FEV / FVC lung capacity in patients with advanced / terminal lung cancer.	8/10
Porter et al.^48^ (2019) United States	RCT n = 63; Breast cancer. IG *n* = 43; 56.3 ± 11.6 years. CG *n* = 20; 59.4 ± 11.3 years.	Duration: 8 weeks. Description: 6 subgroups of yoga classes. 8 sessions were carried out per week, lasting 120 min each of them. An evaluation was conducted after the third and sixth month following the date of the intervention.	High satisfaction with the number of the participants on the Intervention Group. Similarly, for the Intervention Group, improvements were obtained in the perception of fatigue (difference IG vs CG 95% CI; −0.6 [−1.7, 0.4]), pain interference (difference IG vs CG 95% CI; −0.6 [−1.5, 0.3]); anxiety (difference IG vs CG 95% CI; −1.1 [−2.8, 0.6]) and depression (difference IG vs CG 95% CI; −0.9 [−2.3, 0.4]).	Yoga has beneficial effects on patients with advanced breast cancer, improving pain control, sleep quality, functional capacity, and mindfulness. Likewise, it helps reduce fatigue and anguish.	8/10
Schink et al.^47^ (2018) Germany	RCT n = 131; Cancer heterogeneity. IG *n* = 96; 60.3 ± 13.1 years; CG *n* = 35; 59.1 ± 11.6 years.	Duration: 12 weeks. Description: 2 workouts per week, including 2 rest days between each session for muscle recovery. 12 min are required for each session initially. Increase of 2 min/ week, up to a total of 20 min. Per workout. Programme of resistance / strength exercises.	The results showed a significant increase for the electrostimulation training group, for the variables of physical function and performance status (*p* < 0.05). Even though there was an improvement, it was not significant for the training group, the variables of quality of life, fatigue and blood parameters.	Electrostimulation physical exercise helps to improve physical condition and performance status when patients with advanced cancer are getting a palliative chemotherapy treatment.	8/10
Schmidt et al.^39^ (2018) Germany	RCT *n* = 67; Breast cancer. Strength Group (SG) *n* = 21; 53 ± 12.55 years; Aerobic Group (AG) *n* = 20; 56.4 ± 10.15 years. CG n = 26, 54.4 ± 11.19 years.	Duration: 12 weeks. Description: strength and aerobic training programme, along with a control group. The AG performed 2 sessions / week of 60 min. Each training, with a cycle ergometer. The SG performed on guided force machines, obtaining first the 1 RM of each of the muscle groups.	Decrease in immune T CD3 cells, TCR αβ and CD4, NK cells, and B CD19 cells 12 weeks after the start of chemotherapy and physical exercise intervention (SG *p*: 0.046; RG: *p* = 0.001; CG: *p* = 0.001). However, the reduction of T and δ cells, and of CD8 T cells was lower in the resistance training groups and the control group, compared to the resistance training group (SG: −10.93% p: 0.13; AG ‐ 16.89% *p* = 0.04; CG: −4.00% *p* = 0.41).	Strength and endurance training no longer suppresses the immune system. Studies with a larger and more representative sample are needed to determine the impact of physical exercise on the immune system in patients with breast cancer during chemotherapy.	8/10
Schuler et al.^45^ (2017) Germany	RCT *n* = 70; Cancer heterogeneity. Group A: *n* = 24; 53.00 ± 17.99 years; Group B: *n* = 23; 53.57 ± 17.72 years; Group C: *n* = 23; 51.91 ± 17.22 years.	Duration: 12 weeks. Description: An individual training programme was provided. Furthermore, it could be implemented at home. Group A was a control group. Group B and C were the intervention groups. In addition, there were rehabilitation sessions at home for Group C.	There are no significant differences for: 6MWT (*p* = 0.29); General fatigue: (*p* = 0.42) and for Severe fatigue. However, a significant longitudinal change was found for Group C in the second evaluation (*p* = 0.017) and in the third one (*p* = 0.006). Significant decrease for Mental fatigue: (*p* = 0.03) in exercise Group B.	Severe fatigue in advanced cancer patients is reduced when patients exercise. This study describes the impact of outpatient physical exercise in patients with advanced cancer.	8/10
Dhillon et al.^28^ (2017) Australia	RCT *n* = 111; Lung cancer. Sample: IG, *n* = 56, 60 years; CG, *n* = 55, 64 years.	Duration: 6 months of supervised duration. Description: 8 sessions / week, at an intensity of 3 MET h. / week. Aerobic physical exercise programme. They were given a pedometer, a physical activity diary, and a notebook with exercises.	No significant differences were obtained for any of the study variables: fatigue, quality of life, symptomatology, physical or functional state or survival.	Patients with stage III and IV with advanced lung cancer can participate in palliative physical exercise therapy, although it does not improve their quality of life and fatigue.	8/10
Tsianakas et al.^62^ (2017) Great Britain	RCT *n* = 42; Cancer heterogeneity. CG: *n* = 21, Men *n* = 10; 66.2 ± 10.2 years; Women *n* = 11; 58.00 ± 11.6 years. IG: *n* = 21 Men *n* = 11; 65.2 ± 11.7 years; Women *n* = 10; 60.00 ± 12.2 años.	Duration: 12 weeks. Description: The intervention consisted of a self‐initiated walking group that performed 30 min on alternate days.	General fatigue: there are no significant differences between the different groups and there are no significant differences between groups for quality of life.	Physical exercise had a great popular reception and brought social benefits to its participants. The intervention requires further exploitation and exploration to obtain meaningful data on the patients under study.	8/10
Ligibel et al.^21^ (2016) United States	RCT *n* = 101; Breast cancer. CG: Women *n* = 53; 50.7 ± 9.4 years. IG: Women *n* = 48; 49.3 ± 9.6 years.	Duration: 16 weeks. Description: 150 min of moderate‐intensity aerobic activity are at home included in the physical exercise programmed. The patients had a permanent in‐person and telephone follow‐up.	Bruce's treadmill test showed no significant differences between the groups. Physical activity self‐assessments did not show significant differences in quality of life between groups.	Physical activity did not demonstrate to have significant improvements in the patients with metastatic breast cancer. Due to the benefits of physical activity in women with an early stage breast cancer, it is necessary to explore new alternatives to see if they gain quality of life and reduce symptoms.	8/10
Henke et al.^49^ (2014) Germany	RCT *n* = 29; Lung cancer. IG: *n* = 18 CG: *n* = 11	Duration: 3 cycles of chemotherapy. Description: aerobic training, 5 workouts / week); strength, 2 workouts / week, as well as breathing exercises	Significant differences in the Barthel index in IG *p* = 0.041. 6MWT: significant differences in IG *p* < 0.05. Muscle strength and dyspnea: significant differences in IG *p* < 0.05. Going upstairs: significant differences in IG *p* < 0.05. Quality of life: it would improve in IG in some of the parameters of the scale: physical functioning *p* = 0.025; hemoptysis *p* = 0.048; peripheral neuropathy *p* = 0.050; cognitive functioning *p* = 0.050.	Aerobic and strength exercise have a positive impact on health. They improve quality of life, physical functioning, neuropathy, hemoptysis, cognitive functioning and independence. In addition, the sensation of dyspnea, strength and endurance also improve. Lung cancer patients undergoing chemotherapy should receive a physical activity intervention as a complementary therapy.	8/10
Jensen et al.^43^ (2014) Germany	RCT *n* = 21; Gastrointestinal cancer. SG: n = 11, Men *n* = 7; 63.0 ± 9.9 years; Women *n* = 4; 52.3 ± 13.3 years. AG: *n* = 10 Men *n* = 3; 61.5 ± 16,4 years; Women *n* = 7; 46.0 ± 8.5 years.	Duration: 12 weeks. Description: aerobic physical exercise and strength 2 workouts / week. SG 60% ‐80% of 1RM, 2–3 sets, 15–25 reps. AG on a cycle ergometer, sessions of 45 min at 60% of the maximum HR, rising to 70% ‐80% from week 5.	Aerobic capacity in cycle ergometer– No significant differences were obtained. Muscle strength (1RM) ‐ there was a significant increase in the SG (leg muscles *p* = 0.001; biceps *p* = 0.017; back *p* = 0.048). Quality of life—significant increase in AG *p* = 0.045. Fatigue ‐ Significant decrease between groups *p* = 0.003 (SG *p* = 0.004; AG *p* = 0.03) Sleep duration ‐ There was a significant increase for both groups *p* < 0.028.	Physical exercise in patients with advanced gastrointestinal cancer receiving palliative chemotherapy improves their physical capacity, such as their muscle strength. These improvements result in an improvement in the general quality of their lives, as well as in the improvement of the duration of sleep and fatigue.	8/10
Cheville et al.^46^ (2013) United States	RCT *n* = 66; colorectal and lung cancer. IG, n = 33, 63.8 ± 12.5 years; CG, n = 33, 65.5 ± 8.9 years.	Duration: 8 weeks. Description: Strength training. The first 2 weeks the patients did 10 repetitions / sets / muscle group. The following weeks they did 15 repetitions. Aerobic training consisted of a 20‐min walk recorded with a pedometer. It was supervised by the professionals with two calls a month.	The intervention group had significant differences due to increased mobility (*p* = 0.01); reduction of fatigue (*p* = 0.02); increase in the sleep quality (*p* = 0.05).	Aerobic and strength exercise, supervised and performed at home, properly planned and structured, improves mobility, fatigue and quality of dreams in patients with stage IV of colorectal and lung cancer.	8/10
Cormie et al.^40^ (2013) Australia	RCT *n* = 20; prostate cancer. IG, *n* = 10, 73.1 ± 7.5 years; CG, *n* = 10, 71.2 ± 6.9 years.	Duration: 12 weeks. Description: The session lasted 60 min, with a 5 min warm‐up and a 10 min cool down, with aerobic and strength exercises (2–4 sets, 8–12 repetitions of the 1RM). Participants who received the intervention were recommended to supplement it with 150 minutes/week of moderate intensity (gait or cycle ergometer).	Muscle strength (1RM): significant improvement in leg extension in the IG *p* = 0.016. 400 meters gait: significant improvement in the IG *p* = 0.010. Intensity of the 6 meters gait speed test: significant improvements in the IG *p* < 0.001. There were no significant differences between groups for: 6 meters fast pace gait, timed and ready, fatigue and quality of life.	Planned and properly structured physical exercise provides benefits for patients with prostate cancer with bone metastases, improving their physical condition, levels of physical activity and lean mass.	10/10
Litterini et al.^41^ (2013) United States	RCT n = 66; Cancer heterogeneity. AG *n* = 32, 62.53 ± 12.83 years; SG *n* = 34, 62.18 ± 14.28 years.	Duration: 10 weeks. Description: 2 supervised training sessions / week from 30 to 60 min. The strength exercise began with 1 muscle set / group from 8 to 15 repetitions according to the tolerance of the patients. For the aerobic exercise group, patients were asked to exercise on a ratio of 10–12 on the Borg perceived exertion scale.	Significant improvements for the short physical performance battery (SPPB) *p* < 0.001 for both groups. Significant improvement for the increase in gait speed *p* = 0.001 and reduction of fatigue for both groups *p* = 0.05.	Exercise has a beneficial effect on the functional improvement of patients with advanced cancer. Aerobic and strength exercise has a positive impact on improving physical performance, as well as on walking speed and reducing fatigue.	8/10
Hwang et al.^44^ (2012) Taiwan	RCT *n* = 24; Lung cancer. Sample: IG n = 13, average age = 61.0 ± 6.3; CG n = 11, average age = 58.5 ± 8 years	Duration: 8 weeks, Description: High intensity aerobic interval training, three times a week on a cycle ergometer. High intensity ranges were 80% VO2peak, with active recovery at moderate intensity 60% VO2peak. The total training time lasted 30–40 min	Significant increase in peak VO2 in IG *p* < 0.005. Significant decrease in fatigue *p* = 0.005 and dyspnea *p* = 0.001 in IG. There were no significant differences for muscle strength (isokinetic) and for quality of life.	Interval aerobic exercise with a cycle ergometer, in patients with non‐small cell lung cancer, improves their physical capacity, reduces fatigue and dyspnea typical of cancer	8/10
Oldervoll et al.^42^ (2011) Norway	RCT *n* = 231; Cancer heterogeneity. IG *n* = 121, 62.6 ± 11,3 years; CG *n* = 110, 62.2 ± 10.7 years	Duration: 8 weeks Description: Supervised aerobic workouts, as well as resistance circuits with 6 stations. The workouts lasted 60 min, two times/week	The results showed a significant increase in the walking test *p* = 0.01, hand grip *p* = 0.05, in the sitting / standing test *p* = 0.05 and amplitude of the walking step *p* = 0.04 for the training group. However, no significant differences were recorded for the fatigue variable for any group	The physical performance of patients with advanced and incurable cancer, with respect to gait, increased strength, and gait amplitude improves. Therefore, aerobic and strength physical exercise is indicated for patients suffering from advanced or terminal cancer.	8/10

Abbreviations: 1RM, 1 Repetition Maximum; 6MWT, 6‐Minute Walk Test; AG, Aerobic Group; CG, Control Group; FEV, Forced Expiratory Volume; FVC, Forced Vital Capacity; HR, Heart Rate; IG, Intervention Group; MET, Metabolic Equivalent of Task; RCT, Randomized clinical trial; SG, Strength Group; SPPB, Short Physical Performance Battery; VO2_max_, Maximum Oxygen Volume; VO2_peak_, Peak oxygen consumption.

### 3.1 Interventions with aerobic training programs.

Regarding the execution of aerobic training programs, 13 of the 15 studies selected used this methodology. The duration of the training sessions varied from 30 to 150 min per session, with 60 min being the standard duration most used by the researchers.^38^, ^39^, ^40^, ^41^, ^42^ These training sessions were structured in three parts: initial warm‐up, with an average duration of 5–10 min, main phase/training, with a duration of 45 min, and a cool down, with a duration of 5 min of stretching exercises.^40^ Similarly, mechanical instruments, such as a cycle ergometer,^40^, ^43^, ^44^ and training disciplines, such as Nordic walking,^38^, ^43^ were widely used by researchers for training. The most notable results obtained from the aerobic training methodology include improvements in both the capacity and physical function of patients as well as a decrease in physical and mental fatigue, an increase in gait intensity, an increase in the quality and quantity of hours of sleep, and improvements in patient autonomy.^41^, ^43^, ^44^, ^45^, ^46^ These changes improve quality of life, highlighting the broad satisfaction of patients who are involved in such training programs and who participate in research.^43^, ^46^

### 3.2 Interventions with strength training programs.

Strength training methodologies were used in 9 of the 15 selected studies, accompanied in turn by aerobic exercise in the vast majority. The duration of strength training was not as precise as that for aerobic training, as it varied depending on the chosen circuit, muscle groups, intensity, sets, repetitions, and rest between sets.^38^, ^42^, ^46^ The most commonly trained muscle groups were biceps, triceps, abdomen, back, quadriceps, and chest, for approximately 12 weeks. The preferred intensity was between 60% and 90% of 1RM (maximum repetition), followed by 2–3 sets, followed by 10–15 repetitions, factors that favor hypertrophy training and muscle mass gain in patients.^39^, ^43^, ^45^, ^47^ The main outcomes obtained using this training methodology were increases in strength and lean mass, a decrease in anxiety, improvements in social life, a reduction in cancer symptoms, and improvements in social life and quality of life.^39^, ^43^, ^45^

Finally, 2 of the 15 articles used specific training methodologies of a specific discipline: yoga and electrostimulation^39^, ^45^ Women with metastatic breast cancer who participated in yoga reported improvements in fatigue and sleep quality and decreased interference from pain or anxiety.^48^ Electrostimulation training^47^ significantly improved the function and physical performance of patients, with positive trends for improvements in fatigue, blood parameters and quality of life.^47^

## 4 DISCUSSION

This systematic review of clinical trials confirms that physical exercise, in addition to being used as preventive therapy in the health system, has been shown to have a positive impact on advanced‐stage cancer patients.^38^, ^42^ Multidisciplinary teams in hospitals and health services are beginning to include regular physical exercise in palliative, dynamic, social, leisure, and recreational therapy to achieve comprehensive patient care.^27^

In the sample analyzed in this systematic review, physical exercise has been shown to be beneficial for gaining muscle mass, reducing fatigue and dyspnea, and improving the quality of life, sleep and autonomy of patients, among others benefits.^38^, ^39^, ^47^, ^49^ The prescription of physical exercise as an adjuvant is made even more necessary by the increase in the number of people who suffer or will suffer from oncological diseases.^10^, ^11^

Strength and aerobic training are the methods mainly chosen by researchers, as shown by related research.^13^, ^32^, ^50^, ^51^, ^52^ Regarding the great heterogeneity of cancers among the population under study as well as their different symptoms, it cannot be concluded that aerobic training is more beneficial than strength training, or vice versa, because both confer benefits to patients.^40^, ^46^ What can be determined is that these training modalities are increasingly used as adjuvant therapies and that disciplines such as yoga and electrostimulation training are being used as variants of physical exercise for patients.^47^, ^48^

Among the different interventions, the training sessions were designed and customized by a multidisciplinary team composed of doctors, psychologists, physiotherapists, and trainers.^43^, ^45^, ^53^, ^54^, ^55^ However, one of the roles that is least involved or that is not reflected within this team is the role of nurses. Within a health team, nurses provide the most frequent patient care; however, few studies noted their participation in the interventions.^42^, ^56^ In addition, just as a coach or trainer is present at the time of physical exercise, a nurse should be in situ to care for patients who experience discomfort or unwanted effects during training sessions.^57^ Notably, the literature indicates that nursing should play a major role in education and support, both for the patient and for family members and caregivers.^27^ In the cases in which nurses do intervene, they mainly serve as case nurses or specialists, playing key roles in determining patient eligibility in clinical trials.^42^, ^57^, ^58^

Regarding the quantification of the training recommended for this type of population, both modalities (aerobic and strength) established an average of between 60 and 90 minutes per training session, divided into a warm‐up, training/main part, and cool down.^40^, ^45^, ^59^ The intensity of the sessions chosen by the research teams was moderate‐vigorous^21^, ^44^ following criteria outlined by the literature, always having to be supervised by health and physical exercise professionals.^28^, ^38^, ^41^

In relation to aerobic training, mechanical elements, such as cycle ergometers, are introduced with the aim of reducing the impact or possible harmful effects on health; poles for Nordic walking were provided in the studies by Jastrzebski et al.^53^ and Cormie et al..^60^ For clinical trials that establish aerobic training as an adjuvant therapy, the main findings include a reduction in dyspnea and fatigue and improvements in the autonomy, physical condition, and quality of life of patients, among others benefits. Therefore, the benefits of aerobic physical exercise in advanced‐stage cancer patients corroborate the results already presented by other studies.^13^, ^58^, ^60^, ^61^ Interestingly, 4 of the 13 studies related to this type of training did not report significant differences for fatigue, a finding that, to a large extent, is due to the heterogeneity of the cancers presented by the sample and the nonpersonalization of training by type of cancer; however, some studies do report a positive trend for this variable.^21^, ^28^, ^45^, ^62^

Regarding strength training, the principles of hypertrophy are introduced with loads approximately 70% of the 1RM, with 2–3 sets and 8–15 repetitions in each set; this training method is the most used in the literature for muscle mass gain.^53^, ^58^, ^63^ The implementation of this hypertrophy training method facilitates physical benefits for the patient, for example, mitigating hypotonia or muscle weakness.^32^, ^36^ Furthermore, high‐volume and high‐intensity hypertrophy training programs can improve the quantity and quality of muscle tissue and increase strength.^63^ Thus, significant increases in strength gain and/or muscle mass were observed in the oncological/metastatic patients in the different selected clinical trials.^40^, ^43^, ^49^ In 3 of the 9 RCTs related to strength training, no significant improvements related to quality of life or a reduction in fatigue were obtained,^40^, ^42^, ^47^ confirming that 66% of the interventions did report benefits to patients with advanced‐stage cancer, as already evidenced in other studies related to this topic.^57^, ^58^, ^61^, ^64^, ^65^ This statement reinforces what has already been seen in aerobic physical exercise trials, i.e., the need to personalize training by type of cancer to maximize the benefits for patients with advanced‐stage cancer.

The personalization and supervision of training by health professionals and trainers was always necessary.^40^, ^41^, ^43^, ^46^ This supervision was associated with achieving the benefits of the training programs, the adherence and satisfaction of the participants, and the avoidance of unwanted effects.

### 4.1 Limitations and strengths

Regarding the limitations that occurred in the preparation and analysis of the results, it is worth noting the small sample of RCTs in the literature. The vast majority of studies on this topic in the literature are quasi‐experimental in nature; therefore, more RCTs on this topic should be conducted. Another limitation reflected in the RCTs analyzed is that there was great heterogeneity of cancers, making it difficult to personalize training for each participant.

This systematic review is current because it presents the knowledge and results of RCTs published in the last 10 years. Notably, although the types of cancers are very different, the training models have always followed the same pattern, as well as their study variables. Finally, the strengths of this study are that it is representative of patients with advanced‐stage disease and that the results analyzed were obtained from several different countries.

## 5 CONCLUSIONS

Aerobic, strength or combined training that is well‐designed, guided, and supervised has a positive impact on advanced‐stage cancer patients. Participation in these aerobic/strength training programs lead to an increase in muscle mass and improve the fatigue/dyspnea, quality of life and autonomy of patients, as well as their quality and quantity of sleep. The training prescription with the reported greatest benefits was aerobic/strength training sessions, lasting 60–90 min, with a medium‐vigorous intensity (walk test, resistance circuits and hypertrophy), structured as a warm‐up, training, and cool down. More RCTs are needed in which training prescriptions are personalized by the type of cancer and in which there are a greater number of participants.

## CONFLICT OF INTEREST

None.

## AUTHOR CONTRIBUTIONS

Sergio Rodríguez‐Cañamero (SRC), Ana Isabel Cobo Cuenca (AICC), Juan Manuel Carmona Torres (JMCT), Diana Patricia Pozuelo Carrascosa (DPPC), Esmeralda Santacruz Salas (ESS), Joseba Aingerun Rabanales‐Sotos (JARS), Tatiana Cuesta‐Mateos (TCM) y José Alberto Laredo Aguilera (JALA).

SRC, AICC, JMCT, DPPC, ESS, JARS, TCM, and JALA were involved in study design and manuscript writing. AICC, DPPC, ESS, JARS, and TCM were involved in data collection. SRC, JALA, and JMCT were involved in data analysis. AICC, JMCT, and JALA were involved in final approval.

## Data Availability

The data will be available at the request of the author.
